# DSAM: A deep learning framework for analyzing temporal and spatial dynamics in brain networks

**DOI:** 10.1016/j.media.2025.103462

**Published:** 2025-01-29

**Authors:** Bishal Thapaliya, Robyn Miller, Jiayu Chen, Yu Ping Wang, Esra Akbas, Ram Sapkota, Bhaskar Ray, Pranav Suresh, Santosh Ghimire, Vince D. Calhoun, Jingyu Liu

**Affiliations:** a Tri-Institutional Center for Translational Research in NeuroImaging and Data Science (TreNDS) - Georgia State, Georgia Tech and Emory, USA; b Department of Computer Science, Georgia State University, Atlanta, USA; c School of Electrical and Computer Engineering, Georgia Institute of Technology, Atlanta, USA; d Los Alamos National Laboratory, NM, USA; e Department of Applied Sciences and Chemical Engineering, Tribhuvan University, Nepal; f Tulane School of Medicine, Tulane University, New Orleans, USA

**Keywords:** Graph neural networks, Temporal convolutional networks, Attention, Resting-state fMRI (rs-fMRI) data

## Abstract

Resting-state functional magnetic resonance imaging (rs-fMRI) is a noninvasive technique pivotal for understanding human neural mechanisms of intricate cognitive processes. Most rs-fMRI studies compute a single static functional connectivity matrix across brain regions of interest, or dynamic functional connectivity matrices with a sliding window approach. These approaches are at risk of oversimplifying brain dynamics and lack proper consideration of the goal at hand. While deep learning has gained substantial popularity for modeling complex relational data, its application to uncovering the spatiotemporal dynamics of the brain is still limited. In this study we propose a novel interpretable deep learning framework that learns goal-specific functional connectivity matrix directly from time series and employs a specialized graph neural network for the final classification. Our model, *DSAM*, leverages temporal causal convolutional networks to capture the temporal dynamics in both low- and high-level feature representations, a temporal attention unit to identify important time points, a self-attention unit to construct the goal-specific connectivity matrix, and a novel variant of graph neural network to capture the spatial dynamics for downstream classification. To validate our approach, we conducted experiments on the Human Connectome Project dataset with 1075 samples to build and interpret the model for the classification of sex group, and the Adolescent Brain Cognitive Development Dataset with 8520 samples for independent testing. Compared our proposed framework with other state-of-art models, results suggested this novel approach goes beyond the assumption of a fixed connectivity matrix, and provides evidence of goal-specific brain connectivity patterns, which opens up potential to gain deeper insights into how the human brain adapts its functional connectivity specific to the task at hand. Our implementation can be found on https://github.com/bishalth01/DSAM.

## 1. Introduction

Resting-state functional magnetic resonance imaging (rs-fMRI) has emerged as a noninvasive technique pivotal for unraveling the intricate function of the human brain. This imaging modality has not only revolutionized our understanding of brain function but has also showcased its potential as a diagnostic tool for assessing brain disorders (Fornito et al., 2015). By capturing intrinsic neural fluctuations during periods of rest, rs-fMRI enables researchers to delve into the dynamic interplay between brain regions, offering a gateway to comprehend functional connectivity patterns. The importance of understanding brain dynamics and related disorders has prompted significant efforts from fields such as traditional machine learning (Farahdel et al., 2021; Ray et al., 2023; Thapaliya et al., 2021, 2023b) and especially deep learning (Heinsfeld et al., 2018; Sapkota et al., 2024; Suresh et al., 2023).

The analysis of rs-fMRI data relies on graph-theoretical metrics to abstract complex brain networks (Azevedo et al., 2022). Dimensionality reduction is a crucial step due to the high-dimensional nature of the data. This can be achieved through (1) temporal dimension reduction by constructing static connectivity matrices; (2) spatial dimension reduction by aggregating voxel-level signals into a predefined region of interests (ROIs) (Wang et al., 2019), and (3) combined temporal and spatial reduction methods like group independent component analysis (Beckmann et al., 2005). These steps help handle the large data volume and address the signal-to-noise ratio challenges (Smith and Nichols, 2018). However, such reduction methods risk losing valuable information. For example, collapsing the temporal dimension oversimplifies brain dynamics, treating it as static, contrary to emerging evidence of continuous connectivity evolution over time (Avena-Koenigsberger et al., 2017; Liao et al., 2017). Additionally, common association measures often rely on linear models, despite the known nonlinear characteristics of brain signals and interactions (Duggento et al., 2018; Goelman et al., 2018).

A better understanding of brain connectivity variations necessitates the mathematical models capable of identifying brain disorder-specific irregularities (Arslan et al., 2018; Ma et al., 2018). Existing methods often compute functional connectivity (FC) matrices independently of the prediction task, limiting their adaptability. A notable attempt by Kim et al. (2021a) computes FC based on learned data representations, yet it lacks adaptability in connectivity estimation. We propose using deep learning (DL) models with learnable weights to gauge goal-specific connectivity matrices. This approach bridges connectivity analysis and predictive modeling, enabling us to uncover nuanced FC patterns in association with brain function and disorders (Mahmood et al., 2022).

Furthermore, FC estimates often follow static or dynamic computation approaches. Static connectivity assumes stationary brain activities, while dynamic FC, on the other hand, reveals recurring patterns that evade static approaches (Allen et al., 2012). Using a static graph-based method to understand dynamic systems can lead to reduced classification performance, as demonstrated by Xu et al. (2020). Notably, Kipf et al. (2018) demonstrates improved outcomes by dynamically reevaluating the learned static graph during testing, emphasizing the importance of leveraging dynamic nature of brain function. Modeling dynamic connectivity is therefore crucial (Yaesoubi et al., 2018). Yet, current methods frequently employs predefined sliding window techniques with window sizes and strides (Armstrong et al., 2016; Damaraju et al., 2014). In this study, we propose to use deep neural networks to present spatial and temporal dynamics and to build a goal-specific FC matrix. This approach addresses the limitations of static methods and predefined sliding window techniques, offering insights into the dynamic nature of brain function.

Graph neural networks (GNN) are the most frequently used DL methods to study FC matrix. Most GNNs learn embeddings of nodes by just considering their neighborhood throughout the whole graph. However, this could be problematic with brain connectome due to the sub-network nature of the brain. Recently, BrainGNN Li et al. (2021) proposed a new GNN architecture that tackled this limitation by proposing a clustering-based embedding method in the graph convolutional layer, which allows nodes to consider their cluster assignment (representing different regions of the brain) while learning embeddings. Similarly, BrainRGIN Thapaliya et al. (2023a) proposed an isomorphism-based clustered convolution with attention-based readouts to improve the prediction power of GNNs. However, the major limitation of BrainGNN and BrainRGIN is that it did not capture the temporal dynamics of the brain. STAGIN (Kim et al., 2021b) employs GNNs with spatio-temporal attention to model dynamic brain networks extracted from fMRI data using dynamic FCs as input to the model. Graph Transformer (Dwivedi and Bresson, 2020) is a recent transformer-based method that uses static FC as features and applies multi-head attention to perform downstream tasks. A much more recent model, Brain Network Transformer (BNT) (Kan et al., 2022b) effectively used transformer-scaled dot product attention and introduced an orthonormal clustering readout function that leads to cluster-aware node embeddings. On the other hand, IBGNN (Cui et al., 2022) initializes a mask based on edges and refines it with sparse control during training to identify top connections and regions for a specific task, aiming to create a task-specific sub-network for interpretation. Offering further comparison, BrainGB (https://braingb.us/) Cui et al. (2023) offers a modular and extensible framework for brain network analysis, focusing on datasets, tasks, and evaluation metrics with an emphasis on reproducibility. While BrainGB provides a comprehensive evaluation platform, its models generally depend on predefined or dynamically computed FC matrices and do not tackle the challenge of learning goal-specific functional connectivity directly from time series data. For example, the Dynamic Brain Transformer with Multi-level Attention for Functional Brain Network Analysis (Kan et al., 2023) integrates static and dynamic FCs using multi-level attention mechanisms but still relies on predefined or partially dynamic connectivity matrices. Similarly, the Transformer-Based Hierarchical Clustering for Brain Network Analysis (Dai et al., 2022) depends on static FC matrices as inputs, limiting its capacity to capture dynamic, task-specific connectivity changes.

We would also like to clarify why working directly with rsfMRI timepoints is inherently challenging and further highlight how our approach differs from methods that do so. Unlike conventional sequential data, rsfMRI time series exhibit spontaneous fluctuations, with each subject’s scan potentially spanning multiple brain states (e.g., alert to drowsy), and no guaranteed temporal correspondence across subjects. This spontaneity makes it difficult to directly extract meaningful patterns from raw, multi-subject time series, prompting many studies to instead rely on precomputed static or dynamic FC metrics, which simplify analysis but often overlook critical temporal dynamics.

Among the methods that use raw rsfMRI time points, FBNetGen Kan et al. (2022a) takes a step forward by generating task-specific connectivity, yet it still relies on precomputed FC during the prediction stage, potentially introducing bias. Similarly, Azevedo et al. (2022) employs raw time points with temporal (TCN/LSTM) and graph blocks, but does not inherently yield a dedicated, task-specific connectivity matrix, limiting the interpretability of underlying brain networks. DICE (Mahmood et al., 2022), while leveraging temporal and self-attention to learn directed connectivity from time series, it does not provide explicit mechanisms for pinpointing critical time points from multi-level feature representations of time series. Furthermore, recent approaches by Huang et al. (2021), Malkiel et al. (2021), and Kim et al. (2023) have also explored the direct use of raw fMRI data. Specifically, Huang et al. (2021) apply deep learning models to raw voxel-level data for classification tasks but do not derive a learned connectivity structure, focusing instead on direct voxel-wise activations. Malkiel et al. (2021) employ self-supervised transformers to create general-purpose fMRI embeddings without extracting a dedicated, task-driven connectivity matrix. Kim et al. (2023) propose a 4D fMRI transformer (SwiFT) that handles large-scale data, focusing on modeling spatio-temporal complexity rather than producing explicit, interpretable connectivity patterns tailored to specific classification goals. In contrast, our model not only circumvents the need for any precomputed FC measures but also integrates explicit task-relevant connectivity learning, identifies key temporal segments through timepoint selection, employs multi-level temporal features for richer representations, and leverages GNN-based spatial modeling. By doing so, we offer a more interpretable, flexible, and clinically meaningful framework for analyzing the brain’s functional connectivity directly from rsfMRI data, bridging the gap between raw data complexity and task-driven insight.

In light of these challenges and opportunities, our paper proposes a new built-in interpretable model, Dynamic Spatio-Temporal Attention Model (*DSAM*), that leverages the power of TCNs, self-attention, and GNNs to unfold the intricate spatiotemporal dynamics of brain directly from rsfMRI timepoints. By doing so, we move beyond previous approaches that either rely on precomputed connectivity or fail to provide explicit interpretability in terms of learned connectivity structure and key temporal segments. Our model integrates these components to yield a more holistic, task-specific understanding of functional connectivity adaptations. Through this approach, we strive to overcome the limitations of conventional methodologies and pave the way for a deeper comprehension of how the human brain learns and adapts its functional connectivity for various complex conditions. We want to point out that although we validate the model via sex classification, it can be used to predict/classify various phenotypes ranging from age, intelligence, and cognitive processes.

Overall, the main contribution of the paper lies in the built-in interpretability and end-to-end training, including (a) **temporal feature extraction directly from raw time series of the brain activity using a novel multilevel adaptation of TCNs,** (b) **shared temporal attention mechanisms to select informative time points,** (c) **node-node self-attention to build goal-specific brain connectivity matrix,** (d) **ROI-Aware GNNs for the spatial dynamics of the brain**.

The remainder of this paper is organized as follows: Section 2 describes our data and preprocessing step, Our *DSAM* model is also presented in Section 2. Experiments and results are discussed in Sections 3 and 4. Finally, Section 5 concludes the paper with a discussion.

## 2. Materials and methods

### 2.1. Data and preprocessing

#### 2.1.1. Human connectome project (HCP) young adult data

HCP rsfMRI data (Essen et al., 2013) is first minimally pre-processed following the pipeline described in Glasser et al. (2013). The preprocessing includes gradient distortion correction, motion correction, and field map preprocessing, followed by registration to T1 weighted image. The registered EPI image is then normalized to the standard MNI152 space. To reduce noise from the data, FIX-ICA based denoising is applied (Griffanti et al., 2014; Salimi-Khorshidi et al., 2014). To minimize the effects of head motion, subject scans with framewise displacement (FD) over 0.3 mm at any time of the scan were discarded. The FD is computed with fsl motion outliers function of the FSL (Jenkinson et al., 2012). There are 152 discarded scans after filtering out with the FD, and 1075 scans are left. For all experiments, the scans from the first run of HCP subjects released under S1200 are used. We use (Schaefer et al., 2017) atlas for brain parcellation, with 100 regions. For each region, the average value is computed for all the voxels falling inside a region, thus resulting in a single time series for each region. After dividing data into regions, each time series is standardized by their score having zero mean and unit variance.

#### 2.1.2. Adolescent brain cognitive development (ABCD) data

ABCD is a large ongoing study following youths from age 9–10 into late adolescence to understand factors that increase the risk of physical and mental health problems. Participants were recruited from 21 sites across the US to represent various demographic variables. Data used in this study are from 8520 children aged 9–10 at baseline, including resting state fMRI image and fluid intelligence, crystallized intelligence, and total composite scores. Data are splitted into training (n = 5964), validation (n = 1278), and test (n = 1278) subsets. There are 4430 male and 4089 female subjects in this study.

We conduct preprocessing on the raw resting-state fMRI data utilizing a combination of the FMRIB Software Library (FSL) v6.0 toolbox and Statistical Parametric Mapping (SPM) 12 toolbox within the MATLAB 2019b environment. The preprocessing encompasses several key steps, namely: (1) correction for rigid body motion; (2) distortion correction; (3) removal of dummy scans; (4) standardization to the Montreal Neurological Institute (MNI) space; and (5) application of a 6 mm Gaussian kernel for smoothing. Similar to HCP dataset, we use (Schaefer et al., 2017) atlas for brain parcellation, with 100 regions.

### 2.2. The overview framework for DSAM

**Fig. 1.**
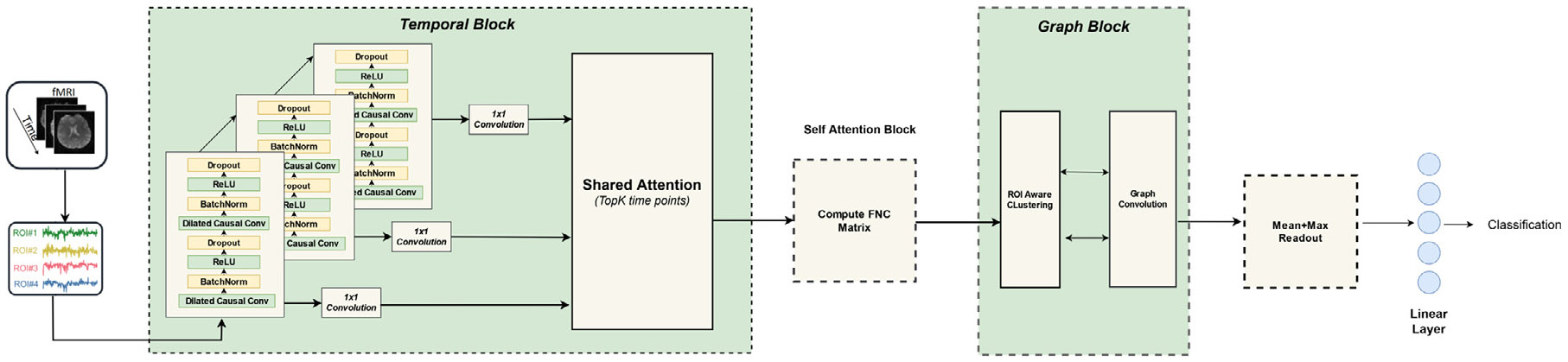
Overall Architecture of *DSAM*. 3 TCN blocks are used to extract the temporal features with different levels of abstraction (low level, medium level, and high level). Temporal attention uses a shared multi head attention module to filter the important time points. Self Attention Block is used to learn the directed FNC matrix, which is used as an input to the Graph Block, followed by Graph Readout and a fully connected layer for classification.

The Dynamic Spatio-Temporal Attention Model (DSAM) as presented in Fig. 1 is designed to effectively capture the intricate spatiotemporal dynamics inherent in complex systems such as brain networks. The DSAM architecture is comprised of several key components that work cohesively to extract informative features from spatiotemporal data and perform classification tasks. It begins with a Temporal Convolutional Network (TCN), capturing temporal dependencies in time series data. The TCN architecture consists of multiple blocks, each aimed at extracting different levels of feature from the input rsfMRI data. Following the TCN, the model incorporates a Temporal Attention Block, utilizing a Transformer-based multi-head attention mechanism, selectively considering informative temporal features in critical time points. Subsequently, the model employs a Self-Attention Block to calculate a learned connectivity matrix across nodes, representing the spatial network as input to the graph neural network model. The graph structure is constructed either dynamically or statically by Pearson correlation of the output from the Temporal Attention Block or original time series. The learned connectivity matrix, is passed as an input to the Relational Graph Isomorphism Network (RGIN) model to capture the spatial dynamics of the data. Finally, the output from RGIN is fed into a linear model for the classification task, leveraging the learned features and interactions within the graph structure. The architecture is further clearly elucidated in the subsections below.

### 2.3. Temporal convolutional networks

Temporal Convolutional Networks form the first component of our spatio-temporal model and are responsible for extracting temporal features from the 1D time-series data of each brain node. It is well documented that TCNs (Bai et al., 2018) can perform equally well or sometimes even better than Recurrent Neural Networks (RNNs) and LSTMs for many kinds of sequential data. Some advantages of the convolutional operator are, for instance, (1) low memory requirement for long input sequences, especially compared to LSTMs and Gated Recurrent Units (GRUs), (2) better parallelization because a TCN layer is processed as a whole instead of sequentially as in RNNs, and (3) easier to train (e.g., it is known that LSTM training can commonly encounter issues with vanishing gradients). TCNs are built on dilated causal convolutions, which are unique 1D filters whose receptive field size quickly grows across the temporal dimension of the input as the network’s depth increases. To maintain temporal order, the padding of the convolution is “causal” in the sense that output at a particular time step is convolved exclusively with elements from earlier time steps from the previous layers.

Formally, given a single ROI time series $t_{i}\in R^{T}$, i is the index of node, *T* is the number of time points, and a filter $f\in R^{K}$, K is the kernel size, the dilated casual convolution operation with *f* at time *t* is represented as:

(1) $$(t_{i}*f)(t)=\sum_{s=0}^{K-1}f(s)\times t_{i}(t-d*s).$$

where d is the dilation factor. When d = 1, a dilated convolution reduces to a regular convolution. Using larger dilation enables an output at the top level to effectively represent a wider receptive field of the input.

We propose a new multi-level adaptation of TCNs that includes three TCN blocks, and each block consists of two temporary convolution layers with multiple channels/kernels, batch normalization, and a dropout. Similar to Bai et al. (2018), we use batch normalization instead of weight normalization because of its stable performance during training. Despite employing the same kernel size across all blocks, the dilation levels progressively increase along with the TCN blocks, as in Eq. (2).

(2) $${\tilde{T}}_{nb}=TCN_{3}(TCN_{2}(TCN_{1}(t_{i},d=1),d=2),d=4)$$

where ${\tilde{T}}_{nb}\in R^{N\times T}$ and *N* is the number of nodes. The three TCN blocks present three levels of features (high-level features, medium-level features, and low-level features) from the time series. At each of the three blocks, we employ distinct 1D convolutions across channels to obtain a single channel representation for that block.

#### 2.3.1. Shared temporal attention block

To identify crucial time points, we introduce a *shared attention* mechanism applied across the features extracted from different levels of TCN. This single attention mechanism filters important time points independently across different blocks while sharing weights. This design choice enforces the attention network to select a similar set of time points across all blocks, enabling a coherent selection of temporal patterns. Subsequently, we apply a top-k approach to filter out unimportant time points.

Initially, the outputs from three TCN blocks, ${\tilde{T}}_{1}$, ${\tilde{T}}_{2}$ and ${\tilde{T}}_{3}$ represented as ${\tilde{T}}_{nb}\in R^{\left(N\times T\right)}$ across all nodes are individually fed to a *shared attention* block to compute the individual attention scores as demonstrated below:

(3) $$K_{T}={\tilde{T}}_{nb}W_{K},\;\;Q_{T}={\tilde{T}}_{nb}W_{Q},\;\;V_{T}={\tilde{T}}_{nb}W_{V}\;\;\text{for}\;nb=1,2,3$$

(4) $$\text{AttentionMatrix}(Q_{T},K_{T})=\text{softmax}\left(\frac{Q_{T}K_{T}^{T}}{\sqrt{d_{K_{T}}}}\right)$$

(5) $$\text{Attention}(Q_{T},K_{T},V_{T})=(\text{AttentionMatrix}\;V_{T})$$

(6) $$\text{Attention}(Q_{T},K_{T},V_{T})=\text{LayerNorm}\;(\text{Attention}(Q_{T},K_{T},V_{T})+{\tilde{T}}_{nb})$$

where $AttentionMatrix\in R^{T\times T}$, $Attention\in R^{N\times T}$, and $W_{Q}$, $W_{K}$, $W_{V}$ are the parameters of the attention block. We implemented multi-head attention (MHA) for this module, followed by *topK* approach to extract the important time points based on *topK* threshold *th* within each block.


(7) $${\hat{T}}_{nb}=topK\;(\text{MHA}({\tilde{T}}_{nb}),th)\;\;\text{for}\;nb=1,2,3$$

(8) $$\hat{T}={⊕}_{nb=1}^{3}{\hat{T}}_{nb}$$

Here, ${\hat{T}}_{nb}\in R^{\left(N\times (T\times th)\right)}$, and ⊕ represents concatenation, thus, the outputs are concatenated to get $\hat{T}\in R^{\left(N\times 3\times (T\times th)\right)}$. The application of the above attention mechanism along the time dimension enables the identification of important time points. By focusing on these crucial time points, we can uncover temporal patterns that contribute to downstream classification tasks.

### 2.4. Spatial self-attention block

Each brain ROI can be considered as a node in the graph, and the FC between ROIs shows how brain regions are linked with each other. To capture the directed FC between brain ROIs, we use a specialized local multihead attention module.

For each node n∈N, a sequence of $\hat{T}$ time point vector, denoted as ${\hat{t}}_{n}\in R^{3\times (T\times th)}$, is fed into the self-attention module to compute the attention matrix $Att_{FC}\in R^{N\times N}$ using specialized local multihead attention. In simpler terms, we are evaluating how each node relates to others over a sequence of time points. Instead of first projecting latent timepoint features into query, key, and value matrices and then dividing them into multiple heads (as in standard attention), we directly divide the input features into multiple heads/sections first and then apply query, key, and value transformations within each section to get the attention weights and average it at the end. This approach helps capture more temporal localized information and demonstrates superior performance in learning dependencies, particularly across time points.

The following set of equations summarizes the process, where T represents transpose and ⊕ represents concatenation.


(9) $$k_{n}^{h}={\hat{t}}_{n}^{(h)T}W{\left(k\right)}^{h},\;q_{n}^{h}={\hat{t}}_{n}^{(h)T}W{\left(q\right)}^{h}$$

(10) $$K_{A}^{h}={⊕}_{n=1}^{N}k_{n}^{h},\;\;Q_{A}^{h}={⊕}_{n=1}^{N}q_{n}^{h}$$

(11) $${Att}_{FC}^{h}=\text{softmax}(Q_{A}^{h}K_{A}^{hT}∕\sqrt{d_{K_{A}^{h}}})$$

(12) $$Att_{FC}=\frac{1}{H}\sum_{h=1}^{H}{Att}_{FC}^{h}$$

Here, $k_{n}^{h}$ and $q_{n}^{h}$ represent the key and query embeddings for each input ${\hat{t}}_{n}^{\left(h\right)}$ within head h. The matrices $K^{h}$ and $Q^{h}$ are formed by concatenating the key and query embeddings across all nodes for each head. The attention matrix ${Att}_{FC}^{h}\in R^{N\times N}$ is calculated for each head. Finally, we take the mean across all heads H to get a single attention matrix $Att_{FC}$, representing the overall connectivity between nodes.

This process improves the model’s ability to estimate directed functional connectivity (FC) and captures more nuanced temporal dynamics between nodes in the graph, which is crucial for downstream classification tasks (e.g., distinguishing between healthy controls and patients). The Directed Connectivity (DC) estimated by the model provides interpretable insights into task-specific node connectivity.

### 2.5. Graph neural network block

We make use of Relational Graph Isomorphism Network (RGIN) along with TopK Pooling to extract the spatial information from rs-fMRI data, which are explained further below.

#### 2.5.1. Constructing a connectivity graph

The brain is spatially divided into *N* ROIs, which represent graph nodes indexed by the set V=1,…,N. The input node features are represented as a matrix $H={\left[h_{1},.\ldots .,h_{N}\right]}^{T}$ where $h_{i}$ is the feature vector of the node $V_{i}$. An edge set *E* represents the functional connections between ROIs with each edge $e_{ij}$ linking two nodes (i,j)∈E. The adjacency matrix $A_{k}\in R^{N\times N}$ is computed is formed by thresholded element $e_{i,j}$ of correlation matrix computes as Pearson’s correlation coefficient between nodes’ feature representation to achieve either fully connected or sparse graph. Correlation matrix is computed in two different ways:

1. Pearson’s correlation coefficient between nodes whose feature representation is the output of shared attention block with dimension $R^{\left(N\times 3\times (T\times th)\right)}$, which is dynamic in nature and changes during training,
2. the Pearson’s correlation coefficient between nodes whose feature representation is the original fMRI data series with dimension $R^{N\times T}$, constant throughout the training.

The input node features, $h_{i}\in R^{1\times N}$, are the output of the self attention block which represents the directed connectivity strengths between node i to all other nodes.

The resultant undirected graph is denoted by the tuple G=(V,E). Let $(H=Att_{FC})\in R^{N\times N}$, $E\in R^{|E|*1}$, and $A_{k}\in R^{N*N}$ symbolize the nodes features, edge features, and adjacency for the graph structure G.

#### 2.5.2. Relational graph isomorphism network (RGIN)

The RGIN convolution, introduced in our recent work (Thapaliya et al., 2023a), offers a novel approach to effectively capture both node and edge features in a graph by merging the concepts of Relational Graph Convolutional Networks (RGCN) and Graph Isomorphism Network (GIN). This method is particularly tailored for understanding brain function, where edge relations signify distinct functional clusters within the brain network. In simpler terms, nodes in the brain are categorized into functional clusters, and different clusters give rise to unique types of edge relations.

Unlike conventional approaches that fix cluster assignments for ROIs, the proposed RGIN model autonomously learns cluster formations. This means that nodes within the same cluster collaborate to achieve optimal performance for the given task. The integration of GIN aids in learning node embeddings for enhanced graph discriminative expression.

In RGIN, the aggregation function of GIN is replaced with that of RGCN, allowing the model to learn distinct mechanisms for different clusters. This design choice is motivated by the fact that GIN employs Multilayer Perceptrons (MLPs) to adapt to the complexity of brain connectivity, while RGCN excels in modeling the sub-network clustering nature of brain connectivity.

The forward propagation function of RGIN is defined as follows:

(13) $$h_{i}^{\left(l\right)}={\text{MLP}}^{\left(l\right)}\left((1+\epsilon^{\left(l\right)})\cdot W_{i}^{\left(l\right)}\cdot h_{i}^{\left(l-1\right)}+\sum_{j\in N_{\left(i\right)}^{\left(l\right)}}W_{j}^{\left(l\right)}\cdot e_{i,j}^{\left(l-1\right)}\cdot h_{j}^{\left(l-1\right)}\right)$$

In standard GNN models, $W_{i}$ and $W_{j}$ are learnable weight matrices. However, in RGIN, they are defined as functions with parameters determined by the cluster assignment of nodes, making it an ROI-aware convolution layer. The model is trained end-to-end with a 2-layered MLP. The parameter $\epsilon^{\left(l\right)}$ is learnable and influences the importance of a node compared to its neighbors.

(14) $$W_{i}^{\left(l\right)}=\theta_{2}^{\left(l\right)}\cdot \text{relu}(\theta_{1}^{\left(l\right)}r_{i}+b^{\left(l\right)})$$

where $W_{i}^{\left(l\right)}$ is a function of the position encoding $r_{i}$, with parameters $\theta_{1}$ and $\theta_{2}$ to build a output of dimension d(l+1)×d(l). ReLU is the rectified linear unit activation function and $b^{\left(l\right)}$ is the bias term in the MLP. We represent each node’s location information, $r_{i}$, using one-hot encoding instead of coordinates, assuming that the ROIs are aligned in the same order for all the brain graphs.

Additionally, we assume that $\theta_{1}^{\left(l\right)}=[\alpha_{1}^{\left(l\right)},\ldots ,\alpha_{N^{\left(l\right)}}^{\left(l\right)}]$, where $N^{\left(l\right)}$ is the number of ROIs in the lth layer, $\alpha_{i}^{\left(l\right)}={\left[\alpha_{i1}^{\left(l\right)},\ldots ,\alpha_{iK(l)}^{\left(l\right)}\right]}^{T}$, $\forall i\in \{1,\ldots ,N^{\left(l\right)}\}$, and K(l) is the number of clusters. In this study, *K* is selected as 7. $\alpha_{i}^{\left(l\right)}$ is the non-negative assignment scores of ROI i to clusters. Assume $W^{\left(l\right)}=[\beta_{1}^{\left(l\right)},\ldots ,\beta_{K(l)}^{\left(l\right)}]$ with $\beta_{u}^{\left(l\right)}\in R^{d(l+1)\times d(l)}$, ∀u∈{1,…,K(l)}, where $\beta_{u}^{\left(l\right)}$ is a basis matrix. Then, we can rewrite Eq. (4) as:

(15) $$W_{i}^{\left(l\right)}=\sum_{u=1}^{K(l)}\alpha_{iu}^{\left(l\right)}\beta_{u}^{\left(l\right)}+b^{\left(l\right)}$$

The incorporation of position encoding $r_{i}$ in $W_{i}^{\left(l\right)}$ is defined using parameters $\theta_{1}$ and $\theta_{2}$ to make the convolution layer specific to clustering. The final forward propagation function of RGIN Convolution can be expressed as:

(16) hi(l)=MLP(l)((1+ϵ(l))⋅(∑u=1K(l)αiu(l)βu(l)+b(l))⋅hi(l−1))+(∑j∈N(i)(l)hj(l−1)⋅(∑u=1K(l)αiu(l)βu(l)+b(l))⋅ei,j(l−1))

This formulation reduces the number of learnable parameters while still allowing a separate embedding kernel for each ROI, contributing to the model’s efficiency.

#### 2.5.3. Pooling layers

Via RGIN convolution, node-wise representations are generated. However, for the prediction task discussed in this paper, which requires graph-level prediction instead of node-level, these node-wise representations must be collected or pooled together. We apply ROI-aware TopK Pooling as it improves interpretability for rsfMRI data, by keeping the most indicative ROIs and removing the noisy and uninformative nodes (Li et al., 2021), along with enforcing sparsity on the network. To select the most indicative ROIs, the choice of which nodes to drop is determined based on the scores of nodes obtained by projecting the node features to 1D via a learnable projection vector $\omega^{\left(l\right)}\in R^{d^{\left(l\right)}}$. The pooled graph $\left(V^{\left(l+1\right)},E^{\left(l+1\right)}\right)$ is computed as follows:

1. Calculate the scores of nodes $s^{\left(l\right)}$ with node feature matrix as follows:
(17) $$s^{\left(l\right)}=\frac{H^{\left(l\right)}w^{\left(l\right)}}{{\left\|w^{\left(l\right)}\right\|}_{2}}$$
2. Normalize the score $s^{\left(l\right)}$ by subtracting its mean and dividing by its standard deviation, yielding final scores ${\tilde{s}}^{\left(l\right)}$:
(18) $${\tilde{s}}^{\left(l\right)}=\frac{\left(s^{\left(l\right)}-\mu (s^{\left(l\right)})\right)}{\sigma (s^{\left(l\right)})}$$
3. Find the top k elements in the normalized score vector ${\tilde{s}}^{\left(l\right)}$ with k- largest values and get their indices as i:
(19) $$i=\text{topk}({\tilde{s}}^{\left(l\right)},k)$$
4. Create the pooled node features $H^{\left(l+1\right)}$ by element-wise multiplying the original node features $H^{\left(l\right)}$ with the sigmoid of the normalized scores and selecting only the elements indexed by i:
(20) $$H^{\left(l+1\right)}={\left(H^{\left(l\right)}\;⊙\text{sigmoid}({\tilde{s}}^{\left(l\right)})\right)}_{i:}$$
5. Create new adjacency matrix as the new pooled graph $E^{\left(l+1\right)}$ by returning the edges between selected nodes i from previous edge matrix $E^{\left(l\right)}$
$$E^{\left(l+1\right)}=E_{i,i}^{\left(l\right)}$$

In this description, ${\left\|\cdot \right\|}_{2}$ represents the $L_{2}$ norm, μ and σ are functions that calculate the mean and standard deviation of a vector, *topK* identifies the indices of the largest *k* elements in a vector, ⊙ denotes element-wise multiplication, and ${\left(\cdot \right)}_{i}$ selects elements in the ith row and all columns.

#### 2.5.4. Readout

Previous studies have proven the effectiveness of the mean and max element-wise pooling operation (Li et al., 2021). For a network, G with l convolution and respective pooling layers, the output graph of the lth pooling block is summarized using a mean and max pooling operation element-wise on $H(l)=[{h(l)}_{i}\;:\;i=1,\ldots ,N(l)]$. The resulting vector is obtained by concatenating both the mean and max summaries. To obtain a graph-level representation, the summary vectors from each layer of the RGIN block are concatenated together, and a 2-layered MLP is used for the final classification.

#### 2.5.5. Loss function

The primary loss function used was a standard Cross Entropy (Zhang and Sabuncu, 2018) loss with additional TopK and Unit Loss similar to our BrainRGIN (Thapaliya et al., 2023a) work. Unit Loss is crucial for ensuring that the learnable node vectors maintain a fixed length of 1, avoiding scaling issues and promoting consistent, meaningful representations, while TopK loss is essential for highlighting the most relevant regions of interest by ensuring that selected nodes have significantly higher scores compared to unselected ones, thus enhancing the interpretability of the model (Thapaliya et al., 2023a; Li et al., 2021). In summary, the total loss $L_{\text{taotal}}$ is a combination of these individual loss components:

$$L_{\text{total}}=L_{\text{CrossEntropy}}+\sum_{l=1}^{L}L_{\text{unit}}^{\left(l\right)}+\lambda_{1}\sum_{l=1}^{L}L_{\text{TPK}}^{\left(l\right)}$$

Where L represents the number of layers in the model, and $\lambda_{1}$ is a hyperparameter that balances the contribution of the TopK Pooling loss. This combination of loss components helps in training the model to achieve good convergence, meaningful node representations, and relevant ROIs selection.

## 3. Experiments setup and model overview

### 3.1. Model overview

Our model as presented in Fig. 1 begins with the utilization of a TCN architecture to extract the temporal dynamics of rs-fMRI time series for each node in the network. Within the three blocks, a kernel of size 7, denoted as *K* = 7, is employed, and the number of channels is 8, 16, and 32 respectively. The dilation factor *d* adopts the form $d=2^{\left(l-1\right)}$, where *l* signifies the block (i.e., l∈{1,2,3}).

Following this, the model transitions into a Temporal Attention Block featuring a Transformer-based *Shared Attention* block (Vaswani et al., 2017). This block incorporates a single-layer attention component with *number_heads* = 4. After extracting the attention scores, we did a hyperparameter search to finalize the optimal threshold for selecting the top-*k* time points (*th*) from the provided range of values: [1, 0.8, 0.6, 0.4, 0.2, 0.1], which resulted in 0.1 providing enough informative feature representations to be used further. The resultant feature set assumes dimensions of $R^{\left(N\times 3\times (T\times th)\right)}$. The *th* as 0.1 or just 10% significantly reduces the time points (using only 360 time points from a total of 3600 time points from three blocks of TCN) and thus the total parameter count later on.

Subsequently, this feature set is passed to the self-attention block to calculate the learned connectivity matrix. The self-attention Block comprises W matrices for key, value, and query operations with the embedding size of 64. The number of attention heads, denoted as *num_attention_heads*, is fixed at 3 (considering concatenated features from three levels). The resulting learned connectivity matrix emerges as a node feature representation with dimensions of $R^{1\times N}$ for each node, which subsequently serves as input to an RGIN model.

Adjacency matrix within the graph structure is constructed using one of two methods: dynamic edges from TCN outputs, and constant edges from original time series. As a result of the hyperparameter search, a fixed threshold of 30% is applied to build a sparse edge representation for both dynamic and constant edge scenarios. We used two layers of RGIN convolution block of sizes [32,32] with a pooling ratio of 0.5, and a 2-layered fully connected layer with sizes [32, 512] with ReLU activation functions in between. The number of clustered communities for RGIN block is set to 7, and the motivation for this comes from the seven functional networks defined by Yeo et al. (2011) to show key brain functional domains. Besides, we fixed the TopK regularizer λ1 to 0.1 to partially force the network to select the same top nodes and also make sure that the selected nodes have different scores than those of the unselected nodes, inspired from BrainRGIN and BrainGNN. In the final step, the output from the RGIN feeds into a linear model for the classification task.

### 3.2. Training the model

The DSAM architecture was implemented using Pytorch (Paszke et al., 2019), and Pytorch Geometric (Fey and Lenssen, 2019) for the specific graph neural network components. The number of nodes was 100 (corresponding to Schaefer Atlas), and the number of node features per node was the number of time points for each node (i.e., 1200). We tested different versions of the architecture with dynamic or constant edges. To evaluate the performance and validity of our model, we conducted proof-of-concept experiments using a widely recognized binary sex prediction task (Jiang et al., 2019; Weis et al., 2019). Our approach employed a 5-fold stratified cross-validation procedure on the HCP dataset. In each fold, we divided the dataset into training and test sets, with the test set comprising 20% of the original data.

The neural network training of our model was performed for 150 epochs, utilizing the Adam optimizer (Kingma and Ba, 2014) and the Cross-Entropy loss function (Zhang and Sabuncu, 2018). The total loss was a combination of CE Loss, Unit Loss and TopK Loss as demonstrated in section 3.1.4 of our BrainRGIN work []. An early stopping mechanism was incorporated, terminating training if the validation loss failed to decrease over 40 consecutive epochs. Additionally, the learning rate was subject to reduction by a factor of 0.1 with a patience setting of 30. We utilized a batch size of 32. Within each random run, we preserved the model with the smallest validation loss. The model exhibiting the lowest validation loss was evaluated on the respective test set. This process was independently executed for each of the five test sets. Metrics derived from these evaluations were subsequently averaged to produce the final results.

We also compared the performance of *DSAM* with popular recent models along with traditional robust ML models as baseline models. The recent baselines includes BrainGNN (Li et al., 2021), BrainRGIN (Thapaliya et al., 2023a), BrainNetCNN (Kawahara et al., 2017), Brain Network Transformer (Kan et al., 2022b), FBNetGen (Kan et al., 2022a), Graph Transformers (Dwivedi and Bresson, 2020) etc. Furthermore, we also compared the performance of our model with a stable deep spatiotemporal model (Azevedo et al., 2022) that also uses TCNs as the initial block to capture the temporal information, but afterward makes use of node and edge models to capture the spatial dependencies. Furthermore, we compared with the baseline models like GAT, and SVM. Based on performance, setting the learning rate of both BrainRGIN and BrainGNN initially to 0.001 and reducing every 30 epochs achieved the best performance for all prediction tasks. For BrainGNN, we used two convolutional layers of size 32, as presented in the original paper (Li et al., 2021), with a fully connected layer of size 512. We utilized the authors’ provided open-source codes for BrainGNN (Li et al., 2021), FBNetGen (Kan et al., 2022a) and Brain Network Transformer (BNT) (Kan et al., 2022b), conducting grid searches to fine-tune essential hyperparameters based on the recommended best configurations. Specifically, for BrainGNN, we also explored various learning rates (0.01, 0.005, 0.001) while for FBNetGen, we investigated how varying hidden dimensions (8, 12, 16) impact the performance. For BNT, we used different hidden dimensions (256, 512, 1024) during scaled dot product attention, along with varying fully connected layers (2 and 3). For Dynamic Brain Transformer (Kan et al., 2023), we used a sliding window of 200 for 1200 time points of HCP, and a window of 24 for the ABCD dataset. The num_heads = 4 was used, and the transformer hidden dimension was tested with (256, 512, 1024) as above. Similarly for THC (Dai et al., 2022), we used clustering size of (20,4) for HCP and (90,4) for ABCD dataset. In the case of BrainNetCNN (Kawahara et al., 2017), we experimented with different dropout rates (0.3, 0.5, 0.7). Regarding GT (Graph Transformer) (Dwivedi and Bresson, 2020), we examined the impact of different number of transformer layers (1, 2, 4) and the number of attention heads (2, 4, 8) over 50 epochs of training. For the training of the model by Azevedo et al. (2022), we used a hyperparameter sweep referencing the parameters suggested in the original paper. The input feature used for this model was also the direct rs-fMRI time points. The hyperparameters used were: batch size = 32, optimizers = [Adam, Rmsprop], dropout a range of values between 0 and 0.5, number of nodemodel layers = [2,3], temporal embedding dimension = [16,32], lr a range of values between 0.01 and 0.00001. The learning rate was scheduled to reduce by a factor of 0.1, same as above with a patience of 30. Other experimental settings were fixed as in the original paper (Azevedo et al., 2022). Besides, we experimented with different numbers of heads = [2,4,6] for GAT, where we also tested with FC features as input features. For SVM, we used Polyssifier (https://github.com/sergeyplis/polyssifier). This tool is widely used to perform baseline model comparisons and has a feature to automatically perform hyperparameter search and identify the best parameters of the model for the highest performance. The input features for SVM was directly the cross-correlation between each of the nodes, which is termed the Functional Connectivity (FC) features.

## 4. Results

In this section, we present the results of our experiments comparing DSAM with the baseline models, and evaluating different forms of the DSAM architecture with varying components. We aim to assess the impact of different architectural choices on the performance of DSAM in capturing dynamic connectivity patterns in brain networks.

### 4.1. Comparison with baseline models

Table 1 provides a comparison of DSAM with baseline models in terms of various evaluation metrics. We report the number of edges, Area Under the ROC Curve (AUC), accuracy, sensitivity, and specificity.

Our DSAM architecture, both in dynamic and constant settings, outperforms the baseline models across all metrics. In the dynamic setting, DSAM achieves an AUC of 83.02%, an accuracy of 82.05%, and strong sensitivity and specificity scores of 85% and 86%, respectively. Even in a constant setting, DSAM maintains competitive performance with an AUC of 81.12% and an accuracy of 80.06%. The comparison with the recent model by Azevedo et al. (2022) baseline reveals significant improvement over existing methods that do not learn the connectivity matrix. Furthermore, we observed stable and comparable performances from baseline models such as BrainNetCNN (Kawahara et al., 2017), FBNetGen (Kan et al., 2022a), and GT (Dwivedi and Bresson, 2020), indicating the reliability of these models. However, the Brain Network Transformer (BNT) (Kan et al., 2022b) exhibited robust performance with stable metrics. These results demonstrate the effectiveness of DSAM in capturing and utilizing dynamic connectivity patterns in brain networks, providing superior classification performance compared to traditional baseline models.

### 4.2. Experiment with the adolescent brain cognitive development (ABCD) dataset

#### 4.2.1. Hyperparameter settings for ABCD dataset

Similar to the HCP dataset, we performed a binary sex prediction task to evaluate the performance and validity of our model. The configuration of the model was same, while the time points of input node was 360. We performed a hyperparameter search to finalize the optimal threshold for selecting the top-k time points (tk) from the provided range of values:[1, 0.8, 0.6, 0.4], similar to HCP. We employed a 5-fold cross-validation procedure. All other hyperparameter settings remain the same.

#### 4.2.2. Results from ABCD dataset

In the context of ABCD, our architecture *DSAM* outperforms most of the baseline models as can be seen in Table 3. In the dynamic setting, DSAM achieves an AUC of 84.12%, an accuracy of 83.27%, an AUC of 81.12%, and an accuracy of 82.06% in the constant setting. Similar to the experiment with HCP datasets, baseline models such as BrainNetCNN (Kawahara et al., 2017), FBNetGen (Kan et al., 2022a), Brain Network Transformer (BNT) (Kan et al., 2022b), and GT (Dwivedi and Bresson, 2020) exhibited stable and comparable performances indicating the reliability of these models in the analysis.

#### 4.2.3. Statistical significance test results of DSAM

The significance tests comparing DSAM (Dynamic Edges) with other baseline models for both the ABCD and HCP datasets on 5-fold cross validation results reveal key insights into its performance. For the HCP dataset, DSAM (Dynamic Edges) exhibited statistically significant improvements over several models, including BrainNetCNN in both AUC (p = 0.0002) and Accuracy (p = 0.0004), BrainRGIN in AUC (p = 0.0104) and Accuracy (p = 0.0000), and Dynamic BT in both AUC (p = 0.0118) and Accuracy (p = 0.0022). Additionally, DSAM outperformed BrainGNN in both AUC (p = 0.0023) and Accuracy (p = 0.0008). While DSAM showed significant differences compared to FBNetGen in AUC (p = 0.0019), it did not show significant differences in Accuracy (p = 0.1893). Comparisons with models like Brain Network Transformer and Transformer-HC showed no statistically significant differences, with p-values exceeding 0.05. For the ABCD dataset, DSAM (Dynamic Edges) showed significant improvements over BrainNetCNN in both AUC (p = 0.0000) and Accuracy (p = 0.0204), as well as over BrainGNN in AUC (p = 0.0001). Moreover, DSAM performed better than N+E (Concat) in AUC (p = 0.0003). However, no significant difference was observed when comparing DSAM to models such as Brain Network Transformer and Dynamic BT. Notably, DSAM showed borderline significance when compared to BrainRGIN in Accuracy (p = 0.0377) and to N+E (Concat) in Accuracy (p = 0.0778), although these results were less conclusive (see Table 4).

### 4.3. Ablation studies

#### 4.3.1. Question 1: How important are each blocks of dsamarchitecture ?

Our ablation studies (performed on the HCP dataset), detailed in Table 2, shed light on the nuanced relationships among various components within the DSAM architecture and their influence on performance metrics, particularly AUC and accuracy. The comprehensive DSAM architecture, encompassing TCNs, Temporal Attention, Self Attention, and GNNs, exhibits notable efficacy with an AUC of 83.02% and an accuracy of 82.05% in dynamic scenarios. When specific components are selectively removed or modified, a discernible decline in performance is observed. For instance, the exclusion of Temporal Attention resulted in a drop of accuracy to 79.52% which as a similar case with 100% time points and with a tremendous amount of timepoints features in the self-attention block with huge performance load. Similarly, leaving out the Self-Attention component and directly using the output of Temporal Attention as input features to GNN, we saw a notable drop in AUC to 73.29% and accuracy to 73.98%. This underscores the pivotal role played by Temporal and Self-attention in extracting essential temporal features and refining the model’s understanding of complex relationships in dynamic settings. Furthermore, the role of GNN (RGIN) in capturing the spatial dynamics is also vividly evident from Table 3, where dropping GNN and using 2-layered MLP directly for prediction after Self-Attention resulted in a significant drop in accuracy.

In summary, our ablation studies highlight the intricate interplay among TCN, Temporal Attention, Self Attention, and GCN within the DSAM architecture. The findings underscore the necessity for a holistic integration of these components to effectively capture dynamic connectivity patterns, showcasing the significance of each element in maintaining the model’s overall performance integrity. These insights provide valuable guidance for refining and optimizing the DSAM architecture for applications involving dynamic learning scenarios.

#### 4.3.2. Question 2: What percentage of original time points are informative for the prediction?

In our investigation of the HCP dataset, we aimed to understand how the selection of time points influences our model’s predictive performance. Through a hyperparameter search across the threshold (*th*) ranges of 10%, 20%, 40%, 60%, 80%, and 100%, we made a surprising discovery: our model achieved its optimal performance with just 10% of the original time points. This selection dramatically reduces the number of features processed by the subsequent self-attention block, with only 120-time points retained per level of TCN from 1200 time points. Considering three levels of TCN, this amounts to a total of merely 360-time points utilized for prediction. Despite this reduction, these selected time points encapsulate crucial information, thanks to feature concatenation occurring at multiple levels within the model architecture. Notably, this reduction in the number of time points also corresponds to a decrease in the total number of parameters, further streamlining our model’s computational load.

#### 4.3.3. Question 3: How does performance alter when we select a larger number of nodes?

We also aim to understand how the number of ROI selections impacts the performance of the model, and if the model learns better with more nodes on the HCP dataset. For this, we used the same atlas (Schaefer et al., 2017) atlas for brain parcellation, but with 200 and 400 regions. As this drastically increases the parameters of the model, we reperformed the hyperparameter optimization for just the dropout selecting a different number of parameters, where dropout of 0.65 for 200 regions and 0.68 for 400 regions gave the best results of 82.15% and 83.01% accuracy. The results reveal that the results are pretty similar, and when optimized, reveal very similar performance.

#### 4.3.4. Question 4: How many blocks of TCN should be used for the best results??

**Fig. 2.**
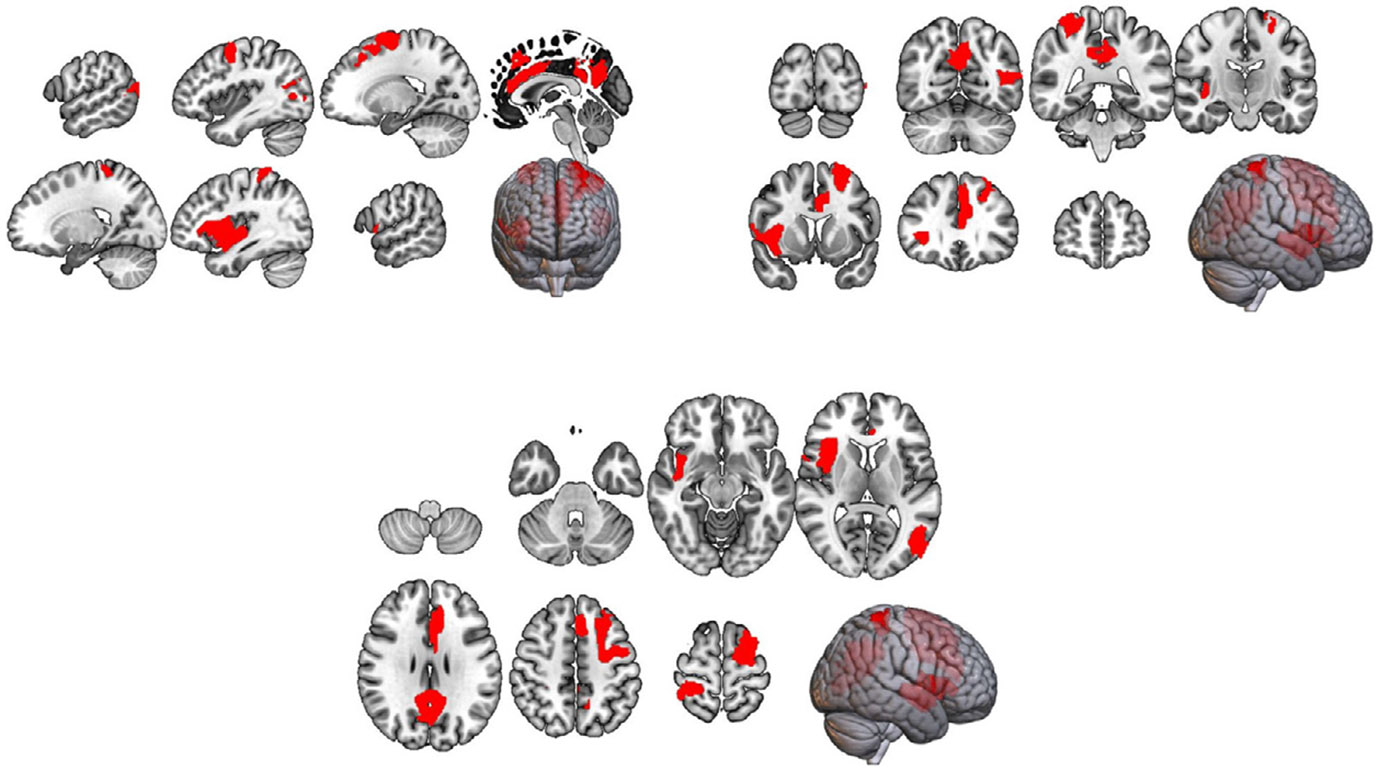
Regions of significant differences between male and female (HCP).

In this section, we answer the idea behind selecting 3 blocks of TCN. The aim of using multiple blocks in TCN is to have diverse representations such that the network can learn a more comprehensive and diverse set of features. Because the dilation increases exponentially, the first block captures low-level temporally local patterns, while subsequent blocks can learn more high-level, large-range, complex features. This hierarchy allows the network to understand the data at different levels of abstraction, potentially improving the model’s ability to capture intricate patterns present in resting state fMRI data. Therefore, we experimented with different numbers of blocks (2, 3, and 4) to see what number of blocks in TCN presented us with the best results. Using the same model configurations, 2,3 and 4 blocks reported an accuracy of 79.12%, 82.05%, and 81.95%, which is an evidence that three-level of feature dilation is enough to capture enough temporal patterns in rs-fMRI data. We did not experiment with blocks larger than 4 blocks because the complexity and parameter of the whole model increased significantly (see Fig. 2).

### 4.4. Directed functional connectivity for sex for HCP dataset

**Fig. 3.**
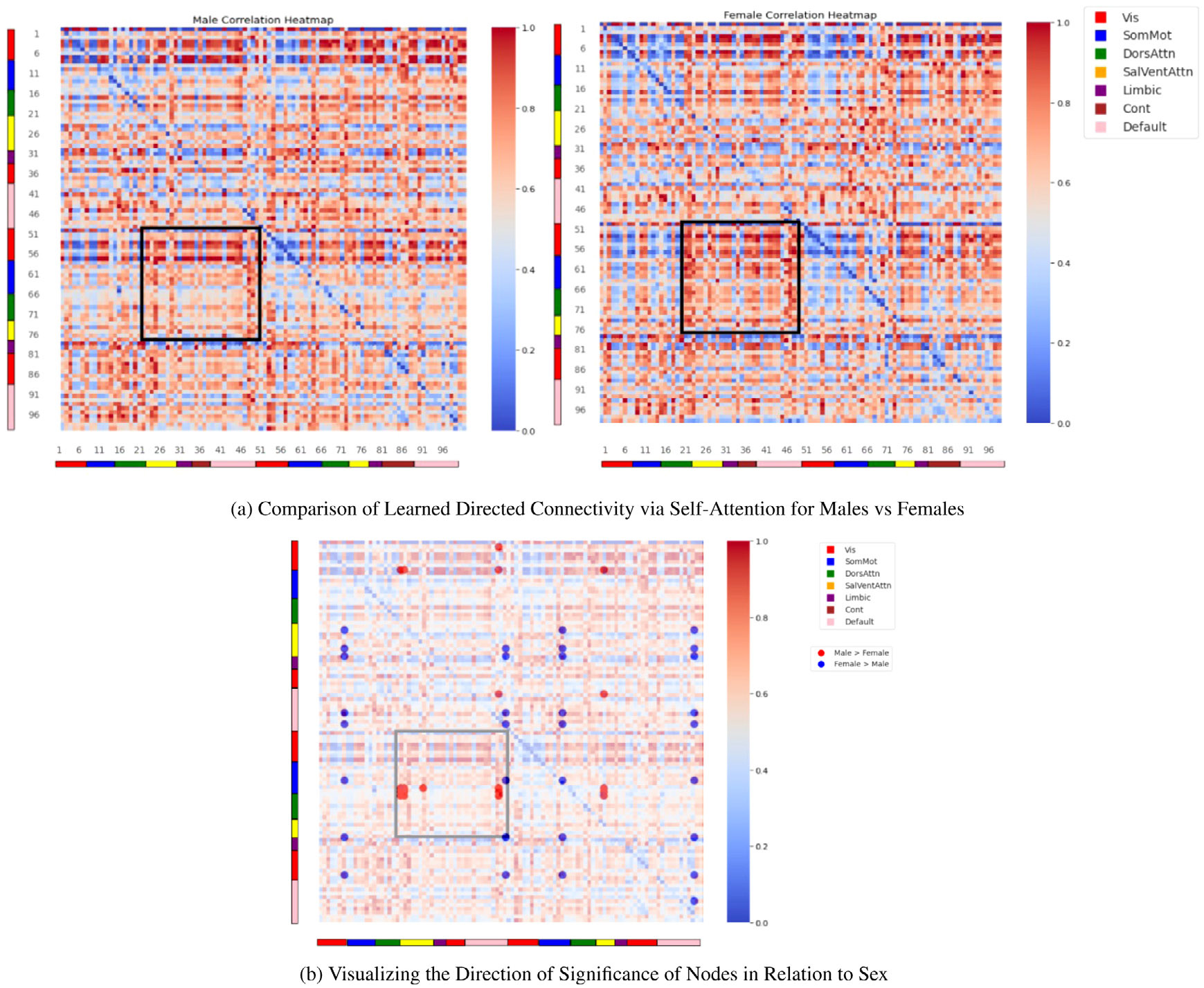
(a) Comparison of learned directed FC between males and females, highlighting regions of interest. (b) Visualization of the direction of significance of nodes concerning sex differences.

Using the model’s inbuilt interpretable power, we plot the learned functional connectivity matrix. The output of the trained self-attention block was extracted from a holdout test set and normalized along the column dimension. The male and female connectivity matrixes were extracted, and the correlation heatmaps are presented in Fig. 3. The 100 ROIs are organized into seven functional networks: *Visual network; Sensorimotor network; Dorsal attention network; Ventral attention network; Limbic network; Control network; Default mode network.*

After extracting the connectivity matrix for both males and females, we performed t-test on all pairs of connectivity between males and female to analyze the results. Multiple comparison with Bonferroni correction was done for both tests.

**Fig. 4.**
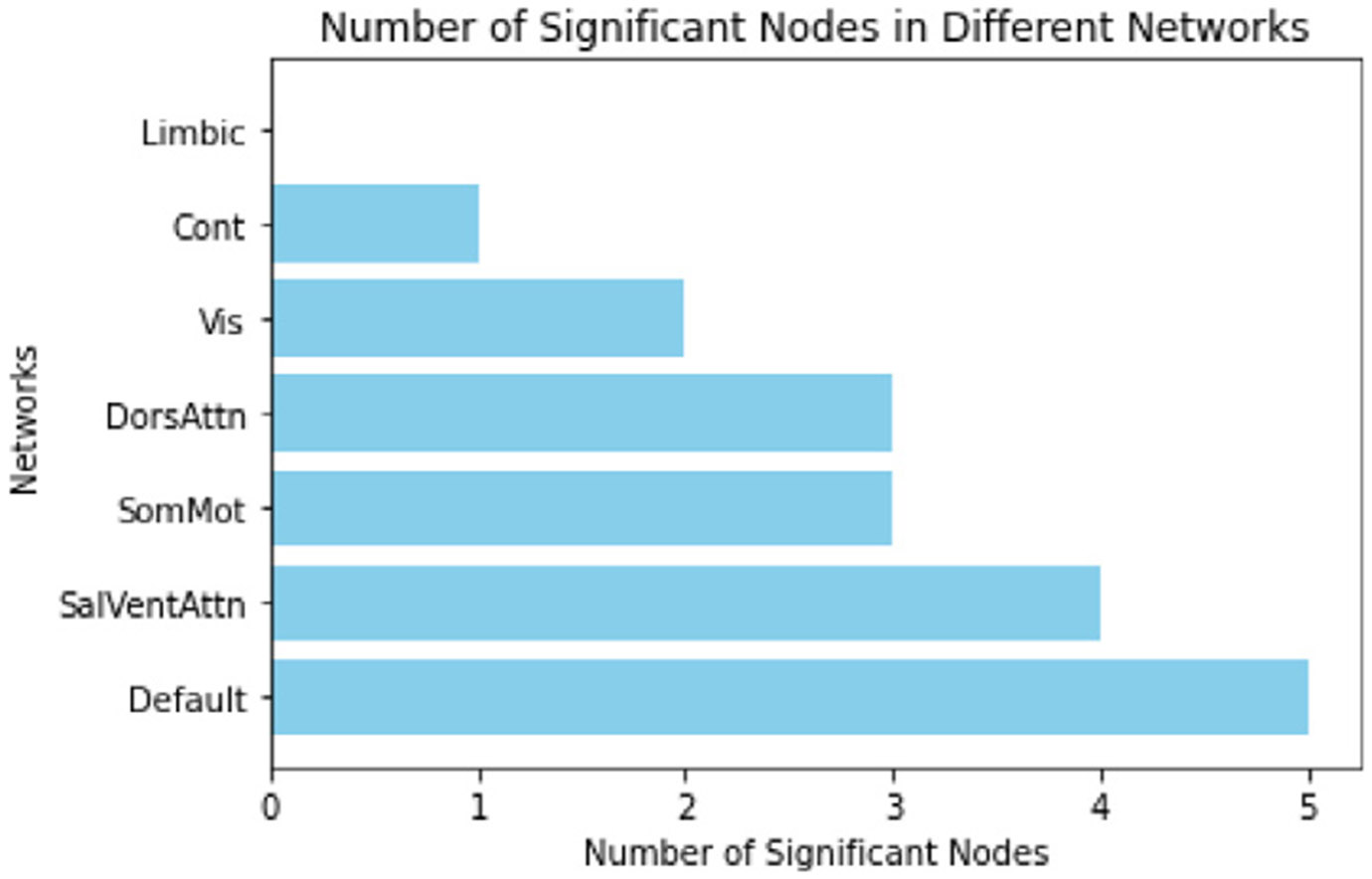
Significant nodes within each network (HCP).

For the t-test between all pairs of 100 nodes, we found connectivity between 52 pairs of nodes (18 nodes involved) to be significant (*p*-value ≤ 5 × 10^−5^). The most involved nodes included the right postcentral gyrus, left calcarine sulcus, left middle frontal gyrus, right and left posterior cingulate cortex (PCC), left cingulum bundle, and right insular cortex. These regions represent key components of the brain involved in somatosensory processing, visual perception, executive functions, default mode network activity, emotional regulation, and social cognition, which we elaborate further in discussion section below. Furthermore, upon grouping the nodes within the 7 connectivity networks, the default mode network had the highest number of nodes (n=5) to be sex differential, whereas the limbic network had zero significant nodes. The complete number of nodes per networks are represented in Fig. 4.

## 5. Discussion and conclusion

In this research study, we have demonstrated the effectiveness of our model, *DSAM*, in capturing the spatiodynamics of the brain and learning connectivity matrices relevant to prediction tasks. Our model leverages TCN, temporal attention, self-attention, and ROI-aware GNNs to comprehensively capture the spatiotemporal dynamics of brain connectivity directly from raw and noisy resting-state fMRI (rs-fMRI) data. Significantly, this approach enhances interpretability and offers a deeper understanding of the mechanisms underlying brain function.

One of the key strengths of our model lies in its direct utilization of raw resting-state fMRI time points, bypassing the need for handcrafted FC features commonly employed by existing models such as BNT, FBNetGen, BrainRGIN, and BrainGNN. This approach represents a departure from conventional methods and presents a more challenging task of seamlessly integrating both temporal and spatial blocks, thus contributing to the model’s “heaviness”. Moreover, our model offers a significant advantage in its ability to learn directed goal-specific connectivity matrices. The directed FC matrices are different from correlation-based static FC (see the Supplementary figure for static FC), suggesting that the model is able to learn new patterns as well as learn effectively critical nodes and connections within the brain for the task. This capability holds immense promise for uncovering relevant phenotypes and advancing our understanding of brain function. Though having differences, we have some similarities in static FC vs learned directed FC for sex where in both t-tests, default mode had the highest number of significant nodes (23 nodes in static FC and 5 nodes in learned FC), and the limbic network had the lowest number of nodes (3 for static FC, 0 for learned FC). This comparison highlights the nuanced differences between static FC and our goal-oriented learned FC, underscoring the relevance and specificity of our approach in uncovering brain connectivity patterns tailored to the task at hand.

In this research study, our decision to focus more on the HCP dataset over the ABCD dataset is rooted in several factors. Firstly, the HCP dataset offers a sample size almost ten times smaller than the ABCD dataset. This smaller sample size makes it more manageable for training and running experiments efficiently, allowing us to explore our model’s capabilities more effectively within the constraints of computational resources.

The results obtained from the interpretation perfectly align with existing research findings in the field of neuroscience. Specifically, research has consistently demonstrated differential activation of the right postcentral gyrus during mental rotation tasks, indicating potential sex-specific cognitive strategies or neural mechanisms (Butler et al., 2006). Furthermore, sex differences observed in regions like the Calcarine have been linked to affective disorders, reinforcing the significance of considering sex-specific neurobiological factors in understanding these conditions (Chaudhary et al., 2023; Tu et al., 2022; Piani et al., 2022). Similarly, investigations into middle frontal volume and connectivity have underscored associations with antisocial personality and autism, emphasizing the importance of sex-specific considerations in elucidating the neurobiological underpinnings of these disorders (Piani et al., 2022; Raine et al., 2009). Moreover, the role of the posterior cingulate cortex (PCC) within the Default Mode Network (DMN) has been highlighted, with findings suggesting significant sex-by-aging interaction and implications for conditions like autism and substance use disorders (Scheinost et al., 2014; Jung et al., 2015; Ritchay et al., 2021). Similarly, investigations into the insula and cingulum, components of the saliency network, have revealed sex differences in connectivity patterns, particularly in the context of autism spectrum disorders, further emphasizing the relevance of sex-specific neural mechanisms in understanding these conditions (Cummings et al., 2020). Additionally, sex differences in morphological measures, particularly those related to interoception, provide further insight into the complex interplay between brain structure, function, and the manifestation of cognitive and affective processes (Longarzo et al., 2021). Integrating findings from these diverse brain regions and networks not only enhances our understanding of sex-specific neural mechanisms but also holds promise for personalized approaches to diagnosis and treatment across various neurological and psychiatric conditions.

As we observe the difference in results when using dynamic edge weights as compared to static edge weights, we assume this is because of the graph being adapted with the temporal features over time, which extracts more relevant edge features as compared to using static edges. This difference helps to adjust the graph over time more specifically to the task at hand. As the main focus of the model is to learn goal-specific connectivity of the brain, we also believe having dynamic edges did open more flexibility for the model to be more oriented towards a specific task.

We want to point out the limitations of the model being “heavy” and performance-resource tradeoffs. Fully aware of its computational weights, each component of our model’s architecture is carefully designed to satisfy the task at hand. That said, we also optimized the model for a lighter computation load by utilizing only a fraction of the original time points from the TCN. Specifically, we retain just 10% of the original time points, significantly reducing the computational load and parameter count, reflecting our deliberate efforts to streamline the analysis process while maintaining performance. Despite the computational demands, the performance gains achieved over simpler models warrant focused and merit attention. The decision to employ a simpler or heavier model depends on case-by-case careful consideration of performance-resource trade-offs. Our purpose here is to present a novel model architecture meticulously designed to align with the requirements of the task at hand, modeling temporal–spatial dynamics of brain function for a specific task.

In conclusion, our model represents a significant step forward in computational neuroscience, offering a powerful tool for analyzing and interpreting brain connectivity dynamics directly from raw rs-fMRI data. As a preliminary version of this study, future work will include additional experiments on specific tasks (such as classifying patients with schizophrenia or Alzheimer’s, predicting cognitive ability). Its utility and effectiveness opens up great potential to gain deeper insights into how the human brain adapts its functional connectivity specific to the task.

## Supplementary Material

appendix

## Figures and Tables

**Table 1:** Comparison with baseline models on HCP Dataset for Sex Classification.

Model	AUC	Accuracy	Sensitivity	Specificity
DSAM (Ours) - Dynamic Edges	83.02 ± 1.5	**82.05** ± **0.83**	85 ± 0.8	85.18 ± 0.5
DSAM (Ours) - Constant Edges	81.12 ± 1.7	80.06 ± 0.4	84.12 ± 1.3	84.34 ± 0.8
Brain Network Transformer (Kan et al., 2022b)	82.82 ± 0.7	81.5 ± 0.8	**85.06** ± **1.7**	**85.34** ± **0.4**
BrainNetCNN (Kawahara et al., 2017)	75.21 ± 2.1	74.3 ± 2.31	74.12 ± 2.7	77.34 ± 1.2
FBNetGen (Kan et al., 2022a)	81.12 ± 1.8	80.12 ± 1.8	83.75 ± 1.3	79.34 ± 0.9
BrainRGIN (Thapaliya et al., 2023a)	79.96 ± 2.5	77.51 ± 0.9	84.12 ± 1.3	74.34 ± 0.2
N+E (Concat) (Azevedo et al., 2022)	77.12 ± 1.7	77.01 ± 0.9	79.32 ± 1.3	80.16 ± 1.6
GAT (Veličković et al., 2017)	78.12 ± 3.34	77.13 ± 2.7	68.12 ± 1.4	69.32 ± 1.8
BrainGNN (Kim et al., 2021a)	80.47 ± 1	77.85 ± 1.1	77.21 ± 0.7	78.83 ± 0.7
Dynamic BT (Kan et al., 2023)	81.36 ± 1.5	80.12 ± 1.3	83.12 ± 1.2	83.32 ± 0.9
Transformer-HC (Dai et al., 2022)	**83.21** ± **3.2**	81.76 ± 2.1	84.76 ± 0.6	83.98 ± 0.9

**Table 2:** Ablation studies with different forms of DSAM architecture on HCP dataset.

Architecture	Edges	GCN features	AUC	Accuracy
DSAM (TCN + Temporal Attention+Self Attention+RGIN)	Dynamic	Learned FC	**83.02**	**82.05**
TCN+Self Attention+RGIN	Dynamic	Learned FC	79.52	79.05
TCN+Temporal Attention+RGIN	Dynamic	TCN outputs	73.29	73.98
TCN + Temporal Attention+Self Attention + 2 Layered MLP	–	–	63.29	66.98
RGIN (Only GNN)	Constant	Static FC	77.51	79.96

**Table 3:** Comparison with baseline models on ABCD Dataset for Sex Classification.

Model	AUC	Accuracy	Sensitivity	Specificity
DSAM (Ours)- Dynamic Edges	**85.12** ±**1.5**	83.27 ±1.75	85 ±1.8	86 ±0.9
DSAM (Ours)- Constant Edges	81.12±1.2	82.06 ±1.1	84.12±1.3	84.34 ±0.9
Brain Network Transformer (Kan et al., 2022b)	84.98±2.1	**84.12** ±1.73	**86.32**±**1.9**	**87.12** ±**1.2**
BrainNetCNN (Kawahara et al., 2017)	79.22 ±1.8	78.21±1.3	78.23±1.3	80.43 ±1.1
FBNetGen	82.12±2.1	84.91±1.1	82.90 ±1.4	82.79 ±0.7
BrainRGIN (Thapaliya et al., 2023a)	80.19 ±2.5	78.23±1.6	81.48±0.8	83.19 ±0.6
N+E (Concat) (Azevedo et al., 2022)	80.92 ±1.1	79.26 ±2.2	82.13 ±0.9	83.66 ±1.3
BrainGNN (Kim et al., 2021a)	80.23 ± 2	79.35 ± 1.3	79.34 ± 1.2	80.32 ± 0.9
Dynamic BT (Kan et al., 2023)	84.12 ± 1.8	82.07 ± 0.5	84.37 ± 1.5	85 ± 0.8
Transformer-HC (Dai et al., 2022)	85.02 ± 1.1	84.16 ± 1.5	85.12 ± 1	84.12 ± 1.3

**Table 4:** Statistical significance test results of DSAM (Dynamic Edges) with other models in ABCD and HCP datasets.

DSAM vs Model comparison	ABCD dataset		HCP dataset	
	AUC (*t*-statistic, *p*-value)	Accuracy (*t*-statistic, *p*-value)	AUC (*t*-statistic, *p*-value)	Accuracy (*t*-statistic, *p*-value)
Brain Network Transformer	−1.20, 0.28	−1.30, 0.24	1.51, 0.18	2.27, 0.06
BrainNetCNN	10.57, **0.00**	3.13, **0.02**	8.04, **0.00**	7.23, **0.00**
FBNetGen	1.54, 0.18	−0.29, 0.79	5.27, **0.00**	1.48, 0.19
BrainRGIN	2.24, 0.07	2.66, **0.04**	3.68, **0.01**	11.86, **0.00**
N+E (Concat)	7.50, **0.00**	2.12, 0.08	9.37, **0.00**	11.14, **0.00**
BrainGNN	8.64, **0.00**	0.56, 0.59	5.09, **0.00**	6.27, **0.00**
Dynamic BT	1.36, 0.22	−1.11, 0.31	3.57, **0.01**	5.10, **0.00**
Transformer-HC	−0.03, 0.97	−2.80, **0.03**	−0.66, 0.53	0.70, 0.51

## Data Availability

Data will be made available on request.

## Appendix A. Supplementary data

Supplementary material related to this article can be found online at https://doi.org/10.1016/j.media.2025.103462.
